# High-Yield Production of a Rich-in-Hydroxytyrosol Extract from Olive (*Olea europaea*) Leaves

**DOI:** 10.3390/antiox11061042

**Published:** 2022-05-24

**Authors:** Costas S. Papageorgiou, Paraskevi Lyri, Ioanna Xintaropoulou, Ioannis Diamantopoulos, Dimitris P. Zagklis, Christakis A. Paraskeva

**Affiliations:** 1 Laboratory of Transport Phenomena and Physicochemical Hydrodynamics (LTPPH), Department of Chemical Engineering, University of Patras, 26504 Patras, Greece; papag011@umn.edu (C.S.P.); cmng3454@upnet.gr (P.L.); cmng3484@upnet.gr (I.X.); cmng3405@upnet.gr (I.D.); dimitriszag@chemeng.upatras.gr (D.P.Z.); 2 Institute of Chemical Engineering Sciences, FORTH/ICE-HT, 26504 Patras, Greece

**Keywords:** hydroxytyrosol, phenols, olive leaves, extraction, oleuropein, *Olea europaea*

## Abstract

The aim of the present study was to explore the high-yield production of hydroxytyrosol, a phenolic compound with very high antioxidant capacity. *Olea europaea* leaves were chosen as feedstock as they contain significant amounts of oleuropein, which can be hydrolyzed to hydroxytyrosol. The chosen techniques are widely used in the industry and can be easily scaled up. Olive leaves underwent drying and mechanical pretreatment and extractives were transported to a solvent by solid–liquid extraction using water–ethanol mixtures. The use of approximately 60–80% ethanol showed an almost 2-fold increase in extracted phenolics compared to pure water, to approximately 45 g/kg of dry leaves. Extracted oleuropein was hydrolyzed with hydrochloric acid and the hydrolysate was extracted with ethyl acetate after pH adjustment. This step led to a hydroxytorosol content increase from less than 4% to approximately 60% *w*/*w* of dry extract, or 10–15 g of hydroxytyrosol recovery per kg of dry leaves.

## 1. Introduction

Biomass has been in the spotlight in recent years for its potential to drive the fuel and chemical industry from being petroleum dependent to being renewable by utilizing various biomass feedstocks. The biorefinery concept includes viable technologies to produce chemicals and fuels from the three main components of biomass, namely cellulose, hemicellulose and lignin [1]. In addition to these main components, biomass also contains extractives, which are substances that can be extracted from the plant material with various solvents (e.g., water, ethanol, acetone, and hexane). These can be primary metabolites (e.g., sugars, proteins, and fatty acids) that are crucial for the growth and function of the plant, and secondary metabolites (e.g., phenolics, terpenes, and alkaloids) that can have supplementary roles for the organisms [2].

Secondary metabolites can function mainly as defense tools against insects and fungal pathogens [3] and many of them are bioactive. For that reason, there have been numerous investigations for applications in drug development [4], functional foods and beverage production, and food supplement manufacturing. These extractives have gained a lot of attention in recent years because of many health claims concerning them and because modern societies invest a lot in health-promoting diets and more expensive functional foods. This in turn has sparked economic interest and created a rapidly growing market. The industry of plant extractives has the advantage of usually utilizing agro-industrial or agricultural wastes as feedstocks with very low cost, but it faces challenges in logistics and biomass pretreatment techniques [5].

**Figure 1:**
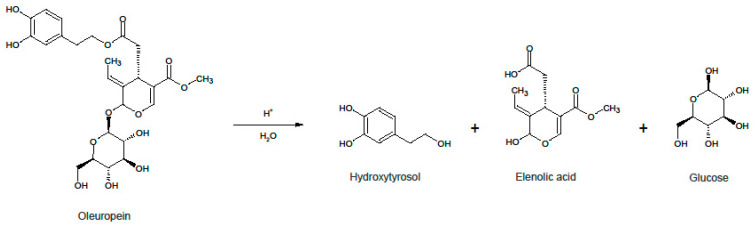
General reaction scheme of the acidic hydrolysis of oleuropein.

Phenolic compounds are a subclass of secondary metabolites and have been associated with various benefits for the human health. In particular, the phenolic compounds in olive trees (*Olea europaea*) are reported to exhibit high antioxidant activity and anti-inflammatory effects, as well as cardioprotective and neuroprotective effects [6,7]. The most abundant phenolic for most olive tree cultivars is oleuropein, a glycosylated secoiridoid with a characteristic bitter taste [8]. This molecule consists of three distinct compounds, namely hydroxytyrosol, elenolic acid and glucose. These can be produced by splitting oleuropein either by enzymatic, alkaline or acidic hydrolysis (Figure 1). Hydroxytyrosol (HT) is a substance with very high antioxidant activity and is mostly responsible for the biological properties of oleuropein. Therefore, it has attracted a lot of research interest in recent years, for deducing its effects on human health [9], but also for finding ways to synthesize it [10,11], or isolate it from olive mill wastes [12,13]. Despite being abundant in olive products, the only product licensed as a novel food in Europe is chemically synthesized HT to be used in fish and vegetable oils. Chemical synthesis is much more convenient and deals with less impurities; so for a commercial process that separates HT from plant material to emerge, the economics of production and the purity of the final extract must become competitive to the synthetic route. 

**Figure 2:**
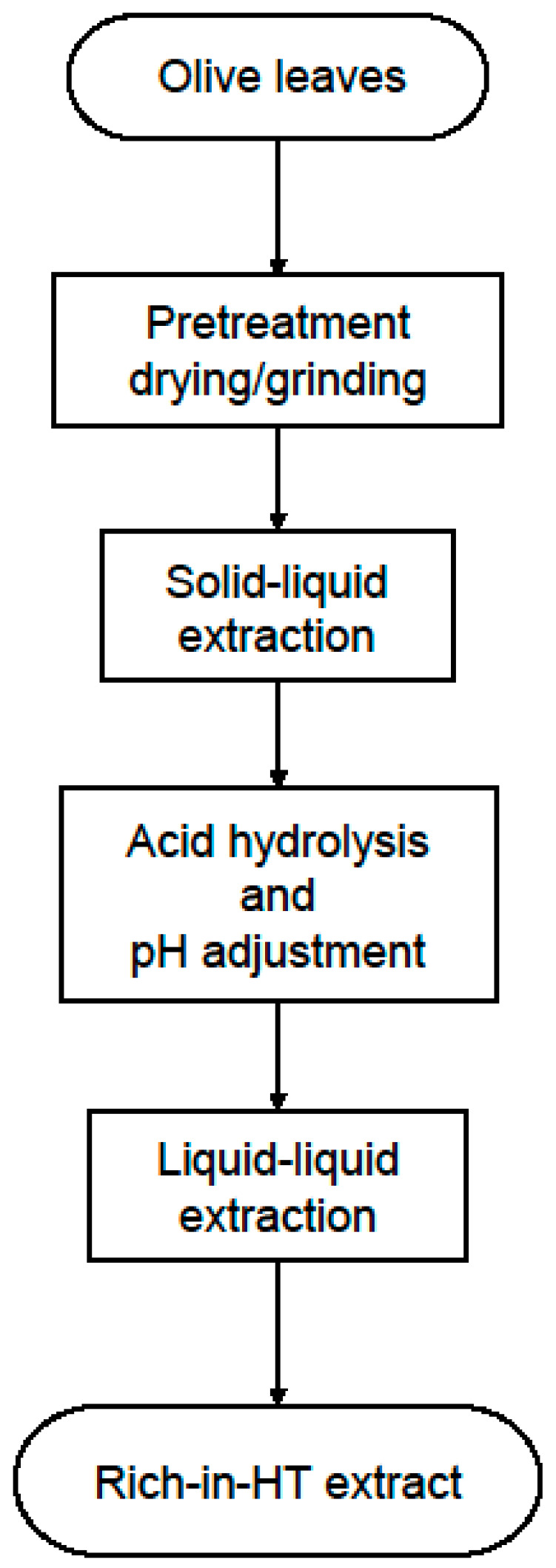
Flowchart of the production of olive leaf extracts that are rich in HT.

In this study, we report a series of treatments to produce a rich-in-HT extract from olive leaves and investigate the effect of different process parameters to maximize the yield. The process consists of four main steps (Figure 2), namely the pretreatment of olive leaves to increase the specific area of the solid material, the solid–liquid extraction with suitable solvents to bring the extractives in the liquid phase, the hydrolysis reaction catalyzed by hydrochloric acid (HCl) to produce HT and pH adjustment after neutralization with sodium hydroxide (NaOH), and liquid–liquid extraction with ethyl acetate to extract HT from the hydrolysate. The organic phase was then concentrated in a rotary evaporator and the final extract (free of ethyl acetate) was analyzed chromatographically to calculate the amount of HT. In most steps, the total phenolic content (TPC) and sugar content are determined spectrophotometrically, and the antioxidant activity of the final extract is measured with the oxygen radical absorbance capacity (ORAC) assay. Finally, we discuss possible ways of bringing the process in the pilot or the industrial scale and how it could be integrated with a biorefinery.

## 2. Materials and Methods

### 2.1. Preparation of Olive Leaves

The olive leaves used in this study were collected during olive harvesting in January 2019 from the “Koroneiki” cultivar in Patras, Achaia, Greece. Small branches were collected after the pruning of the trees was finished, washed with tap water to remove solids and any dirt and pesticide residues, and then placed in a dry room with air circulation and a dehumidifier to remove as much humidity as possible over the course of 2 weeks. After that period, relative humidity was found to be approximately 5%, which was sufficient for pulverization of the olive leaves with a typical blade grinder. Olive powder was then sieved to particles smaller than 0.71 mm and stored in an airtight container at −5 °C for future experiments. For extractions on fresh leaves, these were washed with tap water, drained, and reduced to smaller particles with a blade grinder.

### 2.2. Quantification and Analyses

#### 2.2.1. Quantification of Hydroxytyrosol

**Figure A1:**
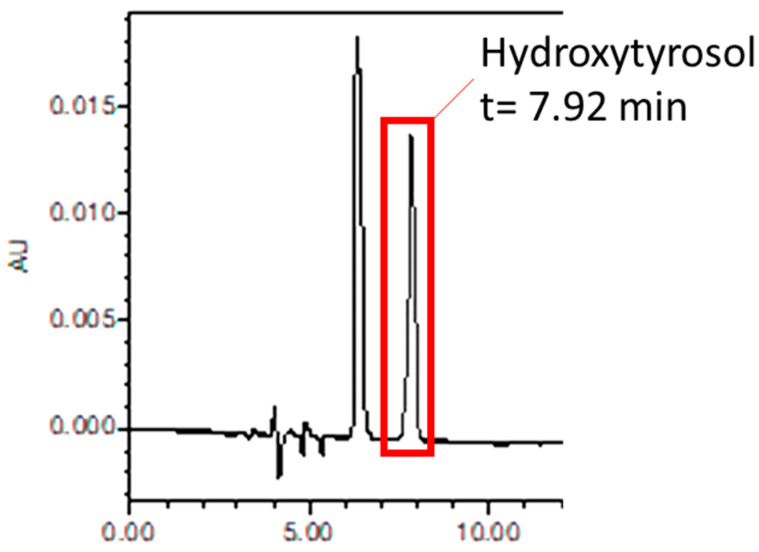
Example of a chromatogram obtained through HPLC of hydrolyzed olive leaf extract.

Hydroxytyrosol was measured with-High Performance Liquid Chromatography (HPLC) on a Waters Alliance 2695 chromatograph. Separation was carried out in a reverse-phase C-18 column (Phenomenex Prodigy, 5 μm, ODS-3 100 Å, 100 mm × 4.6 mm). The mobile phase was 0.1% trifluoroacetic acid (TFA, Sigma Aldrich, St. Louis, MO, USA) in HPLC–MS-grade water (A) by Sigma Aldrich, and HPLC-grade acetonitrile (B) by Sigma Aldrich. The hydroxytyrosol standard was purchased from Sigma Aldrich and its purity was >98%. The chromatographic method was a gradient elution starting at 10% acetonitrile and staying for 10 min and then gradually increasing the organic phase up to 90% acetonitrile over the course of 30 min. The focus was given to hydroxytyrosol quantification, which was eluted at approximately 8 min and therefore the other peaks after 10 min were deemed not important. An example of a chromatogram obtained through HPLC is presented in Appendix A, Figure A1. Error bars correspond to the standard deviation of measurements carried out in triplicate.

#### 2.2.2. Total Phenolic Content (TPC) and Total Dissolved Sugars

TPC was determined spectrophotometrically at 760 nm with the Folin–Ciocalteu reagent (Sigma Aldrich), using gallic acid as standard (Sigma Aldrich) [14]. The phenolic content is expressed as gallic acid equivalents (GAE). Dissolved sugars were also determined spectrophotometrically with a reagent containing l-tryptophan and boric acid (both from Sigma Aldrich) dissolved in concentrated sulfuric acid (Sigma Aldrich) [15]. This method can quantify sugars that can act as reducing agents and since most monosaccharides and oligosaccharides can be mild reductants, it was chosen as a suitable method for this analysis. The calibration standard for this method was α-d-glucose (Sigma Aldrich) and detection was performed at 525 nm. Both spectrophotometric methods gave concentrations of extracted compounds expressed as grams per liter of solvent (g/L). The data presented in this work are expressed as grams of extracted compounds per kilograms of olive leaf powder (g/kg) simply by dividing their concentration in the liquid phase by the solid–liquid ratio. This gives us an overestimate of the extracted material because there is a considerable amount of retention in the solid phase (approximately 30%) but is presented in this way to express the whole amount of extractable material. Error bars correspond to the standard deviation of measurements carried out in triplicate.

#### 2.2.3. Oxygen Radical Absorbance Capacity (ORAC)

Antioxidant capacity was determined with the oxygen radical absorbance capacity (ORAC) assay [16]. For this method, a kinetic fluorescence measurement was performed on black 96-well plates (Greiner Bio-One, F-Bottom, chimney well, non-binding) with a Tecan Infinite 200 microplate reader that was set to 485 nm for excitation and 530 nm for emission. The protocol was approximately 1 h long and during that time temperature was kept constant at 37 °C. For each sample, at least five dilutions were performed and measured to account for errors that are typical in micro assays. The standard used for calibration was Trolox (Sigma Aldrich).

### 2.3. Solid–Liquid Extraction

The solid–liquid extraction took place in a beaker under magnetic stirring or in a jacketed vessel that allowed for temperature control with a heating bath. First, the liquid was poured in the extraction vessel and then the solid material was introduced slowly to ensure good mixing. Different parameters were tested to decide on the best conditions for maximizing hydroxytyrosol quantity and percentage in the final extract. The tested parameters were contact time, solid–liquid ratio, solvent type and temperature. After the extraction was finished, the mixture was filtered under vacuum using 0.22 μm hydrophilic PVDF filters to separate the liquid from the plant material. PVDF was specifically chosen as a low-binding and chemically resistant material to prevent adsorption of oleuropein and to keep its integrity while passing solvents, acids or bases through it. A portion of the filtrate was taken for analysis and the rest was either used for further processing or discarded.

### 2.4. Acid Hydrolysis

Acid hydrolysis with hydrochloric acid (Sigma Aldrich) was performed to hydrolyze the oleuropein molecule and produce hydroxytyrosol. In the experiments where hydro-ethanolic mixtures were used, most of the ethanol was removed from the system prior to hydrolysis because ethanol would interfere later in the liquid–liquid extraction step by increasing the miscibility of the two phases. Decreasing the percentage of ethanol was implemented using a rotary evaporator and condensing the mixture up to 1/4 of its original volume. Then, an aqueous hydrochloric acid solution was added to the condensate and the mixture was boiled under reflux for 15 min. The parameters tested for hydrolysis were acid concentration and reaction time. The reaction was then stopped by immersing the reaction vessel in a cool bath and an aqueous sodium hydroxide (Sigma Aldrich) solution was introduced to neutralize the hydrolysate and adjust the pH at a desirable value. Τhe neutralized solution was filtered under vacuum through 0.22 μm PVDF filters to remove any solids that precipitated during evaporation, hydrolysis and neutralization. A sample from the filtrate was kept for analyses and the rest was used for liquid–liquid extraction with ethyl acetate.

### 2.5. Liquid–Liquid Extraction

Hydroxytyrosol was recovered from the hydrolysate by liquid–liquid extraction with ethyl acetate (Sigma Aldrich). Three equal portions of ethyl acetate were used to extract the target molecule, with each portion being one third in volume compared to that of the initial aqueous phase. Afterwards, the organic layer was washed two times with a saturated sodium chloride (Sigma Aldrich) solution (brine), to remove residual water and impurities from it. Each portion of brine had approximately half the volume of the organic phase. The only parameter that was tested to optimize the efficiency of the extraction was the pH of the aqueous phase. The organic layer was condensed in a rotary evaporator up to a point that it could be retrieved from the round flask and the rest of the solvent was evaporated over the course of two days in a fume hood with absence of light, resulting in a brown viscous liquid with characteristic odor. The extracts resulting from this process were sealed and placed in a freezer at −5 °C to be analyzed later.

Therefore, in this work, the effect of the pH was examined, since it is a parameter that can be altered easily without adding much to the cost of the process in case of scale up. Temperature would be much harder to be examined because in general, the efficiency of this type of extraction is increased by lowering the temperature. Since cooling would greatly add to the cost of a particular set of equipment in case of scale up and since the partition coefficient is large enough for adequate separation, the experiment was chosen to be run at room temperature. 

## 3. Results and Discussion

The above experimental techniques were used to optimize the recovery of hydroxytyrosol from olive leaves. Because a lot of steps are involved in the total process, not every possible parameter was examined; only those that have practical and/or economic importance in the case of scale up.

### 3.1. Pretreatment

The drying of fresh olive leaves was an effective technique, not only because it allowed their pulverization in small particles (<1 mm), but also for their long-term storage without affecting the phenolic composition. Drying is an energy-intensive method; therefore, its benefits must outweigh the added cost for energy and equipment. Moreover, drying conditions should not be too harsh or otherwise the integrity of the bioactive compounds may be compromised. The current literature supports drying as an effective method that increases the extraction efficiency for total phenolics [17,18,19]. These works show that the recovery of total phenolics can be increased by conventional hot air drying even at temperatures as high as 120 °C and also increased recovery in oleuropein when using hot air drying at 105 °C, compared to fresh and freeze-dried leaves [18]. Our results agree with the aforementioned studies, showing a 2-fold increase in extracted phenolics on a dry basis in the case of dried olive leaves compared to fresh ones (Table 1). Sugars saw an increase as well, but not as big as in the phenolics’ case, which can be attributed to phenolics being less available compared to other extractives. Particle size is also responsible for this increase because fresh leaves could not be grinded below 1 cm, while dry leaves could easily reach sizes less than 0.71 mm. Drying has also a positive effect on the preservation of extractives. Dry olive leaves that were grinded and extracted after a storage period of 2 months in an open-to-the-atmosphere vessel, showed a very small decrease in phenolics and sugars in both the aqueous and the hydro-ethanolic extraction (Table 1).

**Figure 3:**
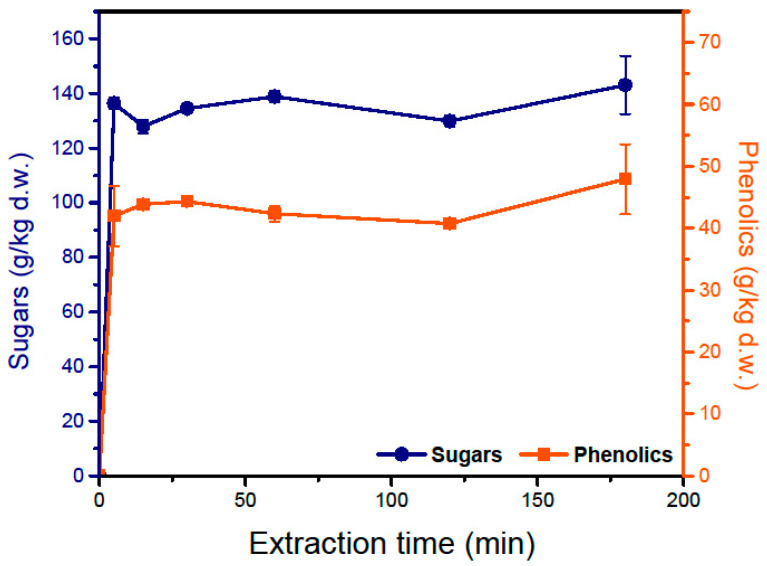
Effect of the duration of extraction on extracted sugars and phenolics; constant parameters: particle size < 0.71 mm, solid–liquid ratio 100 g/L, 40% (*v*/*v*) ethanol as the solvent, and room temperature.

Drying, however, would not have much of an impact without the subsequent grinding of the leaves. Mechanical size reduction techniques are known as viable biomass pretreatment methods, because they increase the specific area available for other treatments (e.g., hydrolysis) and reduce the chain length and crystallinity of the cellulose chains [20]. This is very desirable in this case, because the solvent has access to a larger surface of the solid material and there is less mass transport resistance. Figure 3 shows that when the extraction was performed with fine and dried particles, the extractives were rapidly transported in the liquid phase, reaching a plateau in just 5 min under stirring. In contrast, in a previous work conducted in our laboratory, where fresh leaves were cut into small pieces and extracted, the time for the extractives in the liquid phase to reach a steady value was over 2 h [21]. This huge increase in the extraction’s efficiency further justifies the need for a drying/grinding process step.

### 3.2. Solid–Lliquid Extraction

The examined parameters in this step were the time of extraction, temperature, the solid–liquid ratio and ethanol percentage in the hydro-ethanolic solution. We already discussed the importance of grinding in speeding up mass transport from the solid matrix to the solvent, making time an insignificant parameter after 5 min of extraction under stirring (Figure 3).

**Figure 4:**
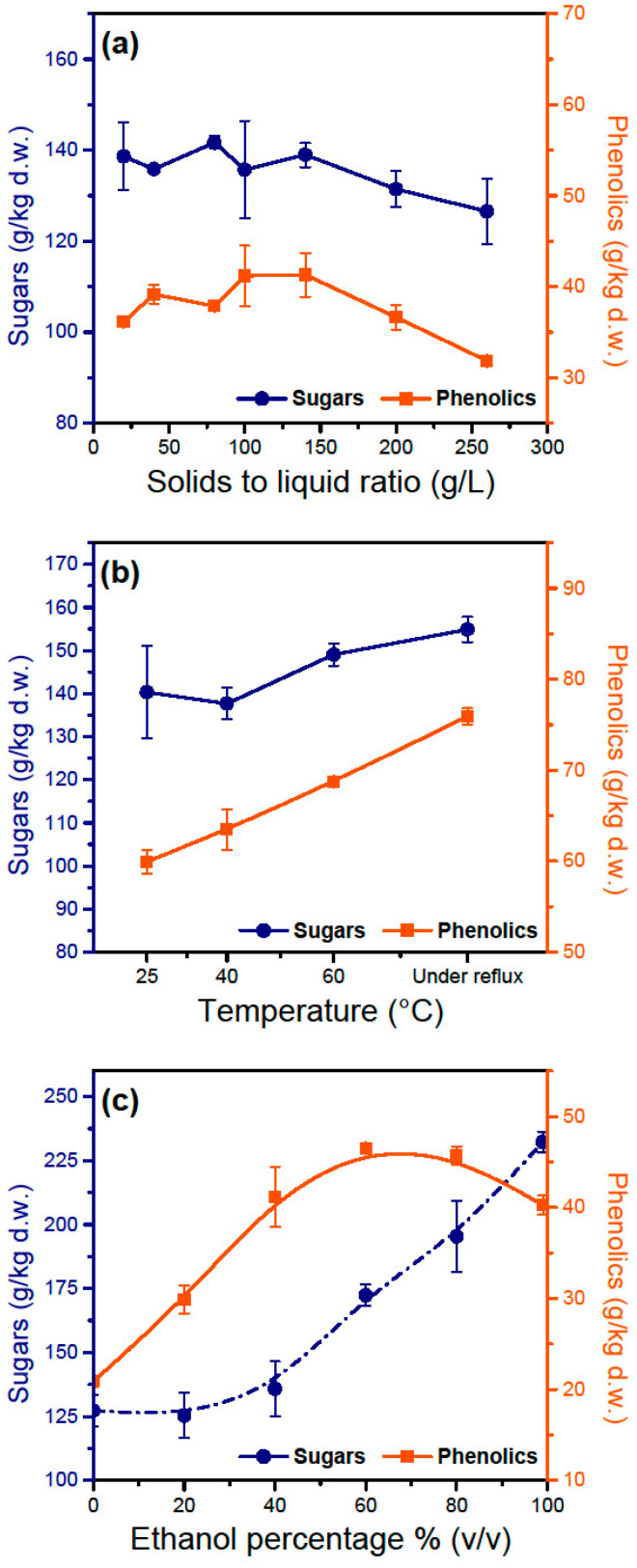
Effect of different parameters on the extraction sugars and phenolics from dry, pulverized olive leaves utilizing conventional solid–liquid extraction. (**a**) Variable: solid–liquid ratio; constant parameters: duration 20 min, 40% (*v*/*v*) ethanol as the solvent, and room temperature. (**b**) Variable: extraction temperature; constant parameters: duration 20 min, 70% (*v*/*v*) ethanol, solid–liquid ratio 100 g/L. (**c**) Variable: ethanol percentage in solvent; constant parameters: duration 20 min, room temperature, and solid–liquid ratio 100 g/L.

Solid–liquid ratio was also not very important in the range it was tested (Figure 4a). The weight of extracted phenolics and sugars per weight of leaf powder (g/kg) remains constant for the most part and only seems to be decreasing after a solid–liquid ratio of 200 g/L. After that point, the solubility of some substances has probably reached its thermodynamic limit and no more material can be transported in the liquid phase without adding fresh solvent. From an economic perspective, increasing this ratio would cut down costs on the solvent’s use and recycling; however, from a practical point of view, using over 250 g/L makes mechanical stirring very challenging due to the high viscosity of the solid–liquid mixture and yield is decreased due to high retention of liquid in the solid matrix. In a real process, the solid powder can either be extracted in a batch reactor with one or two washing cycles to increase efficiency, or in a continuous leaching equipment (e.g., Rotocel). The second option would be the most economical in terms of solvent use and the most efficient, allowing continuous operation.

Temperature had a significant effect on the recovery of phenolics showing almost a 30% increase between 25 °C and boiling point (Figure 4b). Sugars do not exhibit a big increase, probably because they are more easily extractable at low temperatures than phenolics. A valid question is whether temperature has a negative effect on the recovery of oleuropein despite having a positive effect on total phenolics, but this will be discussed later in this study.

Ethanol presence in the solution also seems to play an important role in the maximization of extracted phenolics and especially of oleuropein. A thermodynamic prediction of the activity coefficients of various phenolics in different solvent systems showed that oleuropein exhibits a much larger solubility in ethanol compared to water [22]. Water is a very poor solvent for oleuropein, and some experimental works have shown that its recovery in this solvent is insignificant [23,24]. Pure ethanol also seems a poor solvent for oleuropein extraction but the data are limited [24]. For the water–ethanol system, oleuropein displays the best recovery when a 70:30 (*v*/*v*) ethanol–water mixture is used [23]. The present study agrees with that trend and a maximum for total phenolics was found between 60 and 80% (*v*/*v*) ethanol (Figure 4c). Pure ethanol gave less phenolics and considerably more chlorophyll, giving the solution a dark green tint. Moreover, with increased ethanol percentages, considerably more sugars are released in the solution which may compromise hydroxytyrosol recovery in later steps. 

A comparison should be made with our previous work [21], where it is shown that TPC remains constant with different percentages of ethanol. This can be attributed to phenolic compounds being degraded during the long-term storage at −25 °C. The fact that the phenolic profile remains constant for all ethanol percentages probably means that the surviving phenolics are water/ethanol soluble molecules such as tannins, and those that normally are responsible for the TPC increase were degraded. According to Cifá et al., [23] oleuropein’s recovery is zero for pure water and increases with ethanol percentage; therefore, if oleuropein was present in the frozen leaves, TPC would have increased with ethanol’s concentration. It is shown elsewhere, that freezing processes such as freeze drying (lyophilization) can damage phenolic glycosides but preserve condensed tannins, whereas air-drying preserves phenolics but has a negative impact on tannins [25]. Other works support the idea that freezing processes can have a negative effect on the phenolic profile of the plant material [26,27]. Adding the fact that conventional dying is more economical and has better scalability, freezing or freeze drying would not be a good option in the case of an olive leaf treatment plant.

### 3.3. Recovery of Hydroxytyrosol

**Figure 5:**
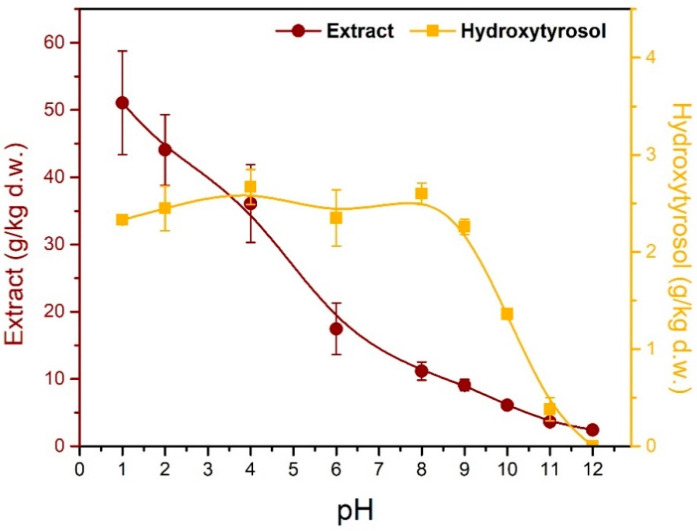
Effect of the pH of the aqueous phase on the quantities of total extract and hydroxytyrosol that were recovered from the organic phase, after liquid–liquid extraction.

The extraction of hydroxytyrosol with ethyl acetate is the last step in the examined process; however, it was deemed necessary to first determine the optimum conditions for its extraction before examining the reaction parameters. An experiment was performed using 3 M HCl_aq._ as the solid–liquid extraction solvent and the mixture was boiled under reflux for 20 min, in order to extract and hydrolyze oleuropein in one step. After that, the hydrolysate was neutralized with NaOH_aq._, and the pH was set to a desired value before extracting its content with ethyl acetate (Figure 5). Previous works have tried to acidify the aqueous medium (olive mill residues), which already has a pH of approximately 5, at pH 2 [28], and 3 [29], before extracting with ethyl acetate; however, in this work, it was found that acidic conditions do not provide adequate purification and the best extracts were acquired at slightly alkaline conditions (pH 8–9).

It can be seen (Table 2) that HCl has a negative effect in the extraction of phenols and sugars when compared to tap water. This could mean that acidic environments are not suitable for phenol extraction or that phenols degrade under acidic treatment. Sugars on the other hand increase with acidic extraction because of hydrolysis reactions that produce monosaccharides and oligosaccharides mostly due to hemicellulose decomposing. After neutralization, these sugars get significantly reduced, which could mean that sugars either got precipitated or converted to sugar degradation products such as HMF (Hydroxymethylfurfural) and furfural. This experiment did not target high yields and the only parameter of interest was the pH, which is an important parameter when having molecules that can act as acids or bases. For example, when an acid needs to remain in the aqueous phase, the pH there needs to be above its pK_a_ in order to let the acid give its proton and take its charged conjugated form. Ions prefer to remain in the aqueous phase, while uncharged molecules of medium polarity will probably be transported to the organic phase. Phenolics are usually weak acids with pK_a_ above 9; and particularly hydroxytyrosol has a pK_a_ value of approximately 9.5. Additionally, the aqueous phase (hydrolysate) contains many different classes of molecules, especially organic acids such as acetic acid and phenolic acids such as p-coumaric and caffeic acid (pK_a_ = 4 and 3.6, respectively). It can be seen (Table 3) that at acidic conditions (pH 1–2), hydroxytyrosol is only 5% of the total extract, but with increasing pH, its recovery remains the same while its concentration in the extract increases. The best conditions seem to be at pH values of 8–9, and after 10, hydroxytyrosol recovery starts to decrease as the pH surpasses the pK_a_ of hydroxytyrosol. At pH 12 no hydroxytyrosol was detected and the extract amount was very small meaning that very few substances passed to the organic phase. Sugars are greatly reduced after extraction for every pH condition, and from being up to 5 times the amount of phenolics in the liquid extract, they became 1/5 of the phenolics in ethyl acetate.

An important parameter that affects the transport of a substance from the aqueous to the organic phase is its partition coefficient. The theoretical partition coefficient for hydroxytyrosol in the two phase system of water and ethyl acetate is large enough for adequate separation with three consecutive liquid–liquid extractions [22].

### 3.4. Hydrolysis of Oleuropein

**Figure 6:**
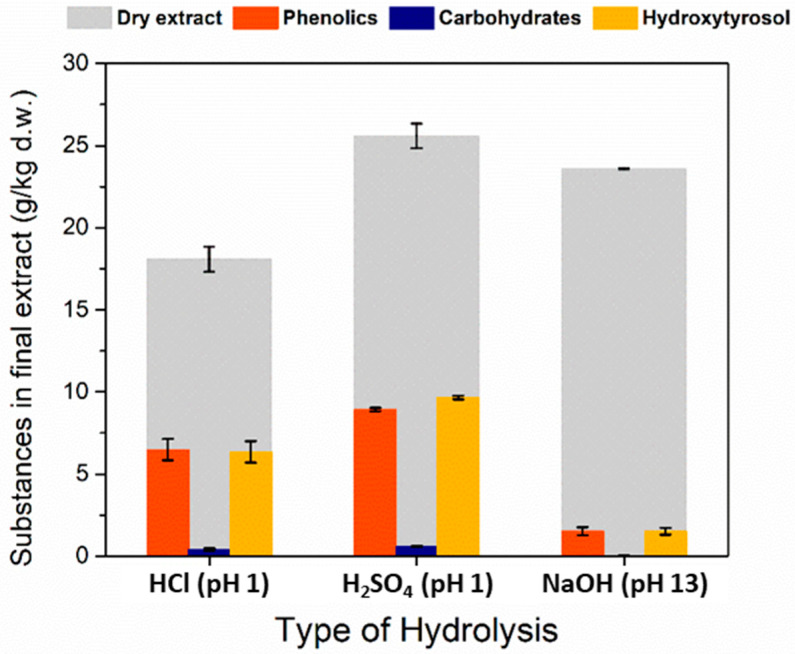
Effect of different catalysts for the hydrolysis of oleuropein. The quantities of substances are measured in the final extract that is claimed after liquid–liquid extraction and evaporation of ethyl acetate. Time of hydrolysis was 15 min.

Reactions can be the most energy-intensive and reagent-consuming steps in a process. It is of great interest in the field of process design to combine adjacent process steps (e.g., reactive distillation and reactive membrane separation). This is called process intensification and it has the potential to decrease capital, operation and maintenance costs. In this case it would be desirable to combine the extraction of oleuropein and its hydrolysis in one step. This could be performed with an acidic or basic solution as extraction solvent. A crude test was performed to examine the efficiency and practicality of each type of hydrolysis. Three separate experiments were performed with every step identical to each other except for the type of catalyst used during hydrolysis. Acidic hydrolysis can be more convenient than alkaline mainly because of the fact that the products are in their acidic form and not their conjugate bases that require an extra protonation step to acquire the substances in their organic form. Moreover, acidic hydrolysis can have more consistent rates of reaction because the protons are regenerated during the reaction and not consumed such as the hydroxyl ions during alkaline hydrolysis. A drawback of the acidic hydrolysis is that the equilibrium may not be that favorable towards the products and probably catalyzing other reactions that decrease the purity and yield of hydroxytyrosol. For the acidic hydrolysis HCl and H_2_SO_4_ were chosen (pH 1) and for the basic NaOH (pH 13) was used. Although the experiments cannot be compared based on the pH values that were chosen because the mechanisms of the two hydrolysis reactions differ, the practicality and purity of product played a big role in choosing the most suitable catalyst for breaking oleuropein in hydroxytyrosol. It can be seen (Figure 6) that the acidic hydrolysis experiments produced greater quantities of phenolics and hydroxytyrosol, whereas the alkaline hydrolysis produced very poor samples in phenolics and hydroxytyrosol. Basic hydrolysis also showed difficulties in processing the material at each stage after hydrolysis. The hydrolysate was viscous, and it was difficult to pass it through a filter and during the liquid–liquid extraction step the two phases separated very slowly and there were many impurities in both of them. Between the two acids, H_2_SO_4_ was considered the most suitable for processes because H_2_SO_4_ is not so harsh on equipment compared to HCl and it can be separated as gypsum after neutralization with Ca(OH)_2_.

**Figure 7:**
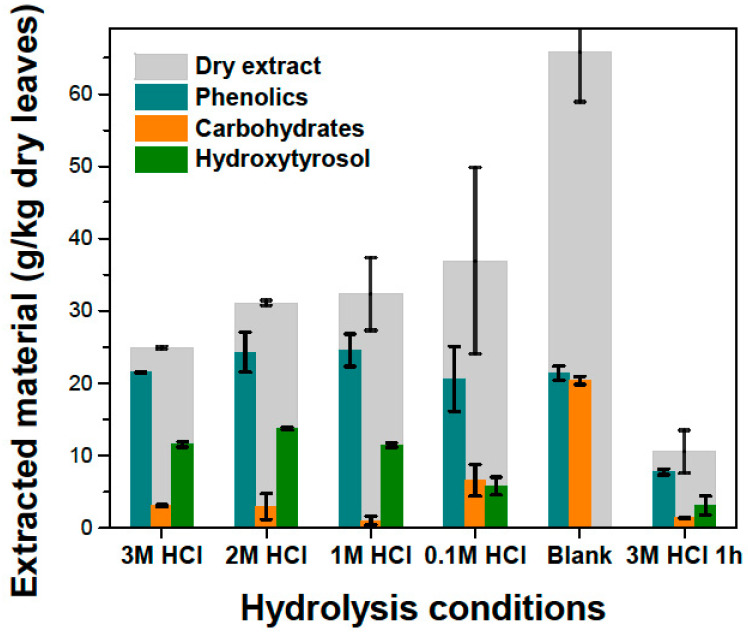
Effect of HCl concentration on the substance profile of the final extract. Blank experiment was performed without any added acid. The experiment with 3 M HCl was also performed for 1 h to examine the effect of hydrolysis conditions on the phenolic profile and mainly hydroxytyrosol.

In the case of HCl, the effect of catalyst amount was studied. It can be seen (Figure 7) that the quantity of extracted hydroxytyrosol is roughly the same when using 3 M, 2 M and 1 M HCl, meaning that hydrolysis is brought to completion during the 15 min of reaction. At 0.1 M HCl, not as much hydroxytyrosol is produced, meaning that 15 min is not adequate for complete conversion. A “blank” experiment with just water was also performed to test the homogeneous hydrolysis when boiling the liquid extract under reflux. In this case, no hydroxytyrosol was detected but the TPC is roughly the same as in the previous conditions. Additionally, there are considerably more sugars, and the total amount of extract is approximately two times that of the previous cases. It seems that the homogeneous hydrolysis of oleuropein-glucoside is insignificant and that oleuropein is extracted in the organic phase instead of hydroxytyrosol. This is supported by the increased sugar content, which is probably the glucose attached to oleuropein; otherwise, if sugars were free in the solution, they would not have been dissolved in the organic phase due to very small solubility in ethyl acetate. TPC, however, remains approximately the same because the Folin–Ciocalteu signal depends mainly on the functional phenolic groups and not the size of the molecule. 

**Figure 8:**
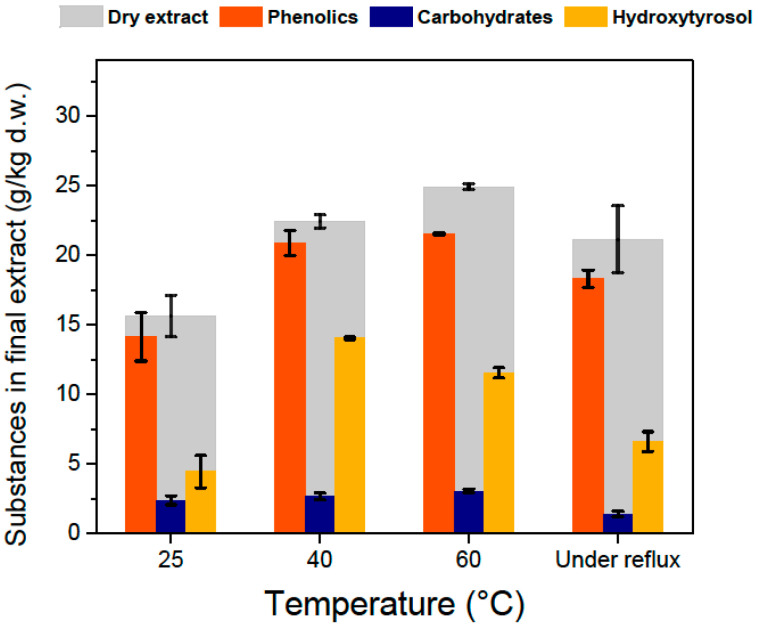
Effect of temperature during solid–liquid extraction, with 70% (*v*/*v*) ethanol, in the substance profile in the final extract. Hydrolysis was performed with 1 M HCl and boiling under reflux for 15 min.

A prolonged hydrolysis experiment for 1 h and 3 M HCl was also performed (Figure 7), where all substances are greatly reduced in the final extract compared to the standard 15-min hydrolysis experiments. This can be attributed to degradation of phenolics at these conditions and shows us that overexposure to heat and highly acidic conditions may compromise the recovery of hydroxytyrosol. This conclusion can also be drawn when looking at Figure 8. When boiling under reflux was utilized for the solid–liquid extraction, the amount of hydroxytyrosol in the final extract was less than in the cases that had lower extraction temperatures. This can be also attributed to oleuropein being partially degraded when exposed at high temperatures. This comes in contradiction with previous studies on olive mill wastes that show that hydroxytyrosol can be extracted with high yields even at high temperatures (180–240 °C) and highly acidic conditions [12], and that oleuropein’s extraction can be maximized at 180 °C [30]. Based on the literature, both hydroxytyrosol and oleuropein present a good stability under high temperatures and the only explanations for our results can be either that hydroxytyrosol and oleuropein react with substances native only on olive leaf extracts, or that the rapid heating with open flame that was used to boil the mixtures was too harsh, creating regions on the glass with temperatures a lot higher than 200 °C. The examination of the effect of temperature that was presented in the solid–liquid extraction section (Figure 4b), was extrapolated to its effect on the concentration of different substances in the final extract, after the evaporation of ethyl acetate (Figure 8). Initially increasing temperature increases the final amount of hydroxytyrosol because more oleuropein is extracted in the hydro-ethanolic solvent. The efficiency in hydroxytyrosol is greatest at 40 °C and decreases a little for 60 °C, contrary to the literature reporting increasing oleuropein extraction efficiencies up to 70 °C. It seems that boiling under reflux has small effect on phenolics, sugars and total extract but has a negative effect on oleuropein, which translates to a reduced amount of hydroxytyrosol in the final extract. This negative effect of increased temperatures cannot be explained easily because it goes against the fact that oleuropein and hydroxytyrosol present good stability at high temperatures. A plausible reason for that in addition to the possibility of the flame as a heating source causing the glass to heat up at really high temperatures, is that at increased temperatures more substances end up in the solvent and some of them may participate in reactions with hydroxytyrosol at elevated temperatures. 

### 3.5. Experiments with H_2_SO_4_ as the Hydrolysis Medium

**Figure 9:**
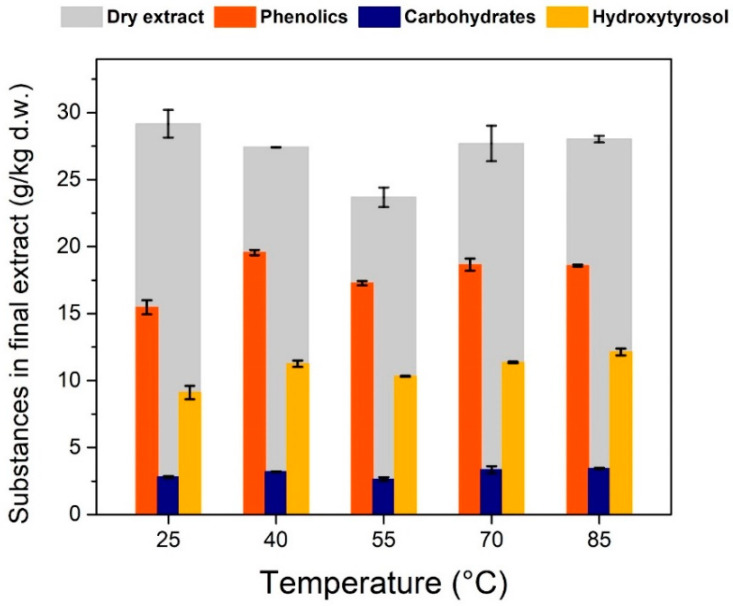
Effect of temperature during solid–liquid extraction, with 70% (*v*/*v*) ethanol, on the substance profile in the final extract. Hydrolysis was performed with H_2_SO_4_ at pH 1 and boiling under reflux for 15 min.

As previously explained, hydrolysis with H_2_SO_4_ is preferred because it has a similar efficiency with HCl but also has process advantages such as reduced corrosiveness and ability to be separated as gypsum after neutralization of the hydrolysate. Therefore, the final set of experiments is done with H_2_SO_4_ and milder hydrolysis conditions in terms of heating to prevent rapid degradation of target substances. The temperature variation experiment during solid–liquid extraction was performed again, but this time with some extra temperature conditions for acquisition of a better trend, and H_2_SO_4_ as the hydrolysis catalyst (Figure 9).

The trend was not in line with that exhibited in Figure 8 and there was a much smaller increase in total phenolics and hydroxytyrosol. However, the amount of extracted compounds was very similar to the maximum values obtained in Figure 8. In Figure 9, it can be seen that the temperature of solid–liquid extraction has little or no effect to the extracted compounds with a slight increase in hydroxytyrosol and total phenolics when temperature increases from 25 to 40 °C. The type of catalyst used for the hydrolysis could have an effect on the final extract’s substance profile, but the mechanism is not clear from our measurements. Temperature, as we saw in Figure 4b, has a positive effect on the extraction of phenolics. In Figure 9, it seems that temperature has no effect at all on the extraction of desirable compounds, but this could have been influenced by the steps that follow after solid–liquid extraction. Some possible reasons for that behavior could be the reactions’ equilibrium during hydrolysis and unwanted reaction taking place, the solubility of the substances during liquid–liquid extraction and their partition coefficients in the water-ethyl acetate system. Another observation from these two figures is the fact that when using HCl as the hydrolysis catalyst, the phenolics and hydroxytyrosol cover a greater percentage of the total material that is extracted from ethyl acetate, than when using H_2_SO_4_.

**Figure 10:**
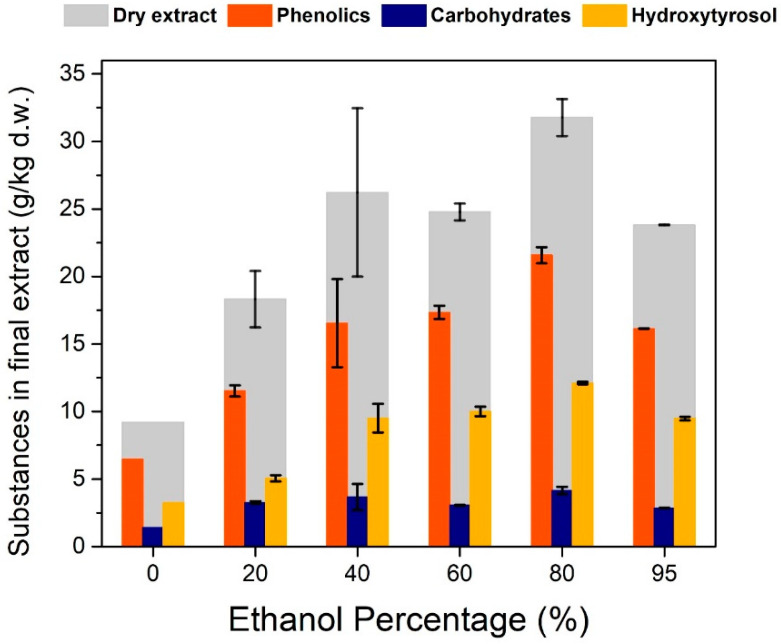
Effect of ethanol’s percentage during solid–liquid extraction, on the substance profile in the final extract. Solid–liquid extraction was performed at 40 °C, hydrolysis was performed with H_2_SO_4_ at pH 1.

The extracted compounds seem to have a greater dependency on ethanol’s percentage during solid–liquid extraction (Figure 10). This comes in accordance with the trend shown in Figure 4c and the maximization of total phenolics happens at 70–80% ethanol. The maximization of hydroxytyrosol happens also at that percentage of ethanol making it clear that for the solid–liquid extraction the optimal condition for recovery is 70–80% ethanol. An economical approach may indicate a smaller percentage of ethanol because of the high cost of ethanol, sacrificing some recovery of hydroxytyrosol. Despite the great differences in the recovery of the extracted compounds at different ethanol percentages, the percentage of total phenolics, sugars and hydroxytyrosol seems to be steady at every condition (approximately 70%, 13% and 40%, respectively). This shows that ethanol plays a big role in extracting target compounds but has no effect on the selectivity. Selectivity here is mostly affected by the pH of the aqueous phase during liquid–liquid extraction. 

**Figure 11:**
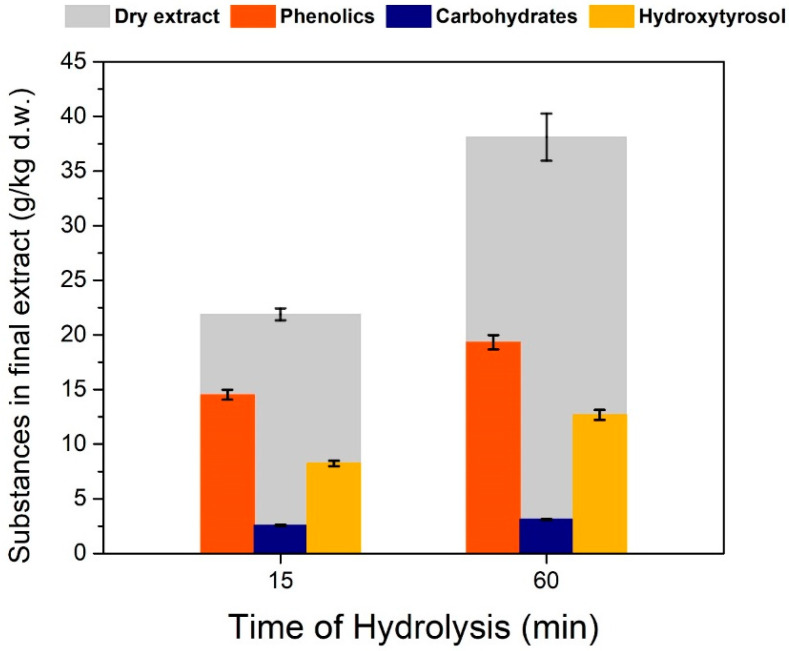
Effect of time of hydrolysis, on the substance profile in the final extract. Solid–liquid extraction was performed at 40 °C, hydrolysis was performed with H_2_SO_4_ at pH 1 and boiling under reflux for 15 min.

Time of hydrolysis (Figure 11) also seems to have a positive effect on the extraction of total phenolics and hydroxytyrosol. At 60 min, the total extract almost doubles from 15 min of hydrolysis, which is probably because of more substances ending up being hydrolyzed and being able to be dissolved in ethyl acetate. However, on the time and acidic conditions of the hydrolysis medium, a more extensive study should be done with kinetic experiments to get a clearer picture of the reaction parameters and the means to design a reactor suitable for oleuropein hydrolysis without many side reactions that would compromise the purity of the final extract. Finally, it should be noted that the experiment in Figure 11, differs to that in Figure 7 in the way that heat was provided to the medium. In Figure 7, heating was performed with open flame hitting the glass directly in order to make the solution boil faster, whereas in Figure 9 heating was done in boiling bath to achieve a more uniform temperature profile. That is probably the reason why in the first experiment target substances have been degraded at 1 h of hydrolysis whereas in the second experiment 1 h gave better results than 15 min of hydrolysis.

### 3.6. Antioxidant Capacity of the Rich-In-Hydroxytyrosol Extract

**Figure 12:**
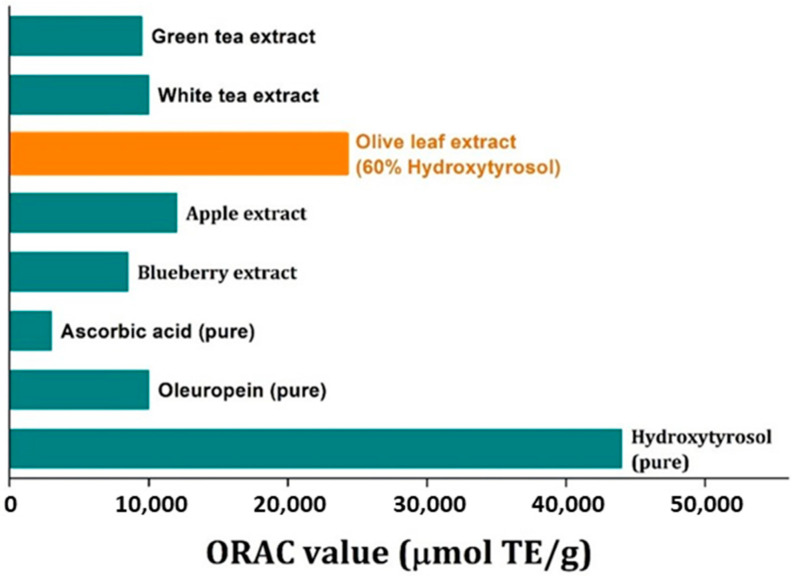
Comparison of the ORAC value of the purest in hydroxytyrosol extract and the ORAC value of commercial extracts and pure substances.

The ORAC method was performed to test the antioxidant capacity of various samples of final extracts. In general, ORAC increased with total phenolics and the amount of hydroxytyrosol in the extract. In Figure 12, the ORAC value of the best extract in terms of hydroxytyrosol recovery and purity is compared with the ORAC value of various commercial extracts (names intentionally not given) and pure substances. It can be seen that the ORAC value of that sample is many times greater than that of ascorbic acid and it is approximately 60% the value of pure hydroxytyrosol, which is logical because the extract contains approximately 60% hydroxytyrosol but there could be contributions from the other phenolics in the extract as well.

### 3.7. Proposed Process Scheme

**Figure 13:**
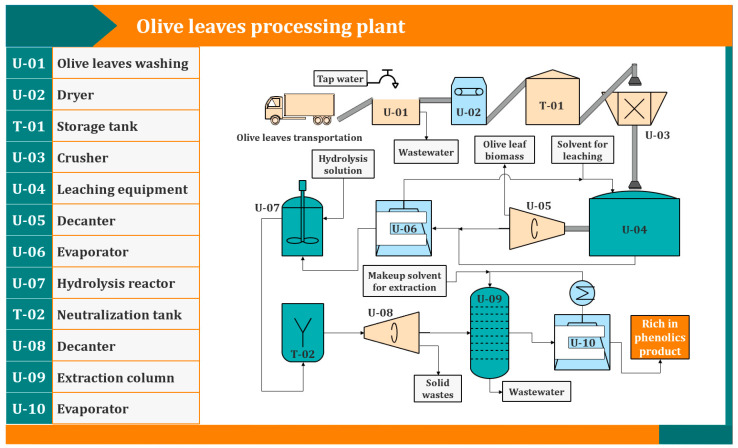
Proposed process scheme for the production of extracts rich in hydroxytyrosol.

The steps that were followed to produce a rich-in-hydroxytyrosol extract are far from optimized and definitely need to be examined more thoroughly. However, the results were very promising and the purity and recovery of hydroxytyrosol was beyond of what was expected with simple physical operations. The authors hope that these results will motivate more research in separating this valuable substance from olive leaves and possibly create incentives for olive refineries and other players in the olive oil sector to invest in such processes in order to produce high added value products. Moreover, the process as it now stands (Figure 13) is fairly complex and some steps can be avoided or merged with others. For example, merging the solid–liquid extraction step with the hydrolysis step, is very desirable to reduce operational, equipment and maintenance costs. 

The described techniques are very common in the industry and can be scaled up very easily, in contrast to continuous chromatographic techniques that require significant capital cost and are very hard to be scaled up.

## 4. Conclusions

In the above work, typical separation techniques were combined with a chemical conversion step to increase the extraction efficiency of hydroxytyrosol from olive leaves. Along the process, many parameters were tested, especially the ones that have a significant impact on practicality and cost. It was found that fine grinding had a significant effect in the efficiency of the solid–liquid extraction, especially for phenolics that were doubled. Fine grinding, however, requires removal of humidity from the olive leaves which is a significant addition to the cost of the process. In order to obtain big yields of hydroxytyrosol at the end of the process, first oleuropein must be extracted efficiently. Hydro-ethanolic solutions gave the best results for oleuropein, 70% (*v*/*v*) ethanol was chosen. Hydrolysis of oleuropein occurred rapidly under reflux even at low concentrations of HCl, such as 0.1 M. Extended reaction times (1 h), however, quickly degraded phenolics and especially hydroxytyrosol, thus, making a more elaborate study on the kinetics of oleuropein degradation vital for better design of the process. pH of the aqueous phase was found to be a very important parameter, and contrary to the literature, slightly basic conditions (pH 8–9) were optimal for high-yield extraction of hydroxytyrosol and minimization of impurities and other phenolics in the final extract. That technique alone managed to increase the amount of hydroxytyrosol in the extract to approximately 60% with the optimal conditions. Reaction with H_2_SO_4_ gave a lower purity in hydroxytyrosol (approximately 40% at best conditions) but may be better overall for the process because of the lower corrosiveness and ability to be removed as gypsum when neutralizing the solution with Ca(OH)_2_. The described techniques are very common in the industry and can be scaled up very easily, in contrast to continuous chromatographic techniques that require significant capital cost and are very hard to be scaled up. We believe that the described process will fit perfectly as an auxiliary process in a biorefinery centered around olive by-products, because the used solids can be processed or used for energy and the added revenue from hydroxytyrosol extracts will further support the economics of the biorefinery.

## Figures and Tables

**Table 1:** Effect of drying and grinding on the extraction of phenolics and sugars and effect of two-month storage on the extractives of dried olive leaves.

Extracted Material	Moisture Content (%)	Solvent	TPC (g/kg d.w.)	Reducing Sugars (g/kg d.w.)
Olive leaves (fresh, cut)	51.2	Tap water	10.74 ± 0.04	118.0 ± 0.0
		50% (*v*/*v*) ethanol	19.23 ± 0.12	114.4 ± 0.8
^1^ Olive leaves (dried, grinded)	5.6	Tap water	20.86 ± 0.64	127.1 ± 6.0
		50% (*v*/*v*) ethanol	39.82 ± 1.16	139.8 ± 17.8
^2^ Olive leaves (dried, grinded)	5.7	Tap water	19.40 ± 0.0	116.0 ± 9.8
		50% (*v*/*v*) ethanol	36.48 ± 0.07	131.6 ± 2.0

Experimental conditions: solid–liquid extraction under stirring; solid–liquid ratio: 100 g/L; temperature: RT; duration: 1 h. Errors represented as standard deviation; ^1^ material processed immediately after drying; ^2^ dried material processed after two-month storage.

**Table 2:** Comparison of water and hydrochloric acid as solvents for solid–liquid extraction.

Solvent Used for Extraction	TPC (g/kg d.w.)	Sugars (g/kg d.w.)
Tap water	58.42	188.80
^1^ 3 M HCl_(aq.)_	43.51	240.92
^2^ 3 M HCl_(aq.)_	43.34	147.56

Experimental conditions: solid–liquid extraction for 30 min under reflux; solids/liquid ratio 100 g/L; ^1^ TPC and sugars measurement performed without neutralization; ^2^ TPC and sugars measurement performed after neutralization.

**Table 3:** Effect of the pH of the aqueous phase on the recovery of different substances.

pH of Aqueous Phase	Extract (g/kg d.w.)	Sugars (g/kg d.w.)	TPC (g/kg d.w.)	Hydroxytyrosol (g/kg d.w.)	Hydroxytyrosol in Extract (%)
1	51.07 ± 7.70	4.01 ± 0.64	19.86 ± 3.46	2.33 ± 0.03	4.57 ± 0.75
2	44.07 ± 5.23	4.01 ± 0.22	17.72 ± 3.46	2.45 ± 0.23	5.56 ± 1.18
4	36.08 ± 5.78	3.91 ± 0.05	17.50 ± 3.61	2.67 ± 0.18	7.40 ± 1.70
6	17.44 ± 3.82	1.74 ± 0.65	10.36 ± 4.15	2.35 ± 0.29	13.48 ± 4.64
8	11.17 ± 1.36	2.07 ± 0.40	8.92 ± 1.78	2.60 ± 0.11	23.23 ± 3.83
9	9.08 ± 0.86	1.34 ± 0.37	5.40 ± 0.52	2.26 ± 0.08	24.84 ± 3.30
10	6.10 ± 0.16	1.39 ± 0.09	3.64 ± 0.05	1.36 ± 0.02	22.33 ± 0.93
11	3.61 ± 0.04	1.62 ± 0.05	2.00 ± 0.21	0.38 ± 0.12	10.40 ± 3.57
12	2.42 ± 0.39	ND *	0.56	ND *	ND *

Experimental conditions: solid–liquid extraction performed with 3 M HCl_(aq.)_ for 20 min; pH adjusted with NaOH_(aq.)_; liquid–liquid extraction performed 3 times with ethyl acetate; organic phase washed two times with saturated NaCl; ethyl acetate evaporated under vacuum. Errors represented as standard deviation; * not detected.

## Data Availability

All of the data is contained within the article.
